# Prediction of Lower Extremity Multi-Joint Angles during Overground Walking by Using a Single IMU with a Low Frequency Based on an LSTM Recurrent Neural Network

**DOI:** 10.3390/s22010053

**Published:** 2021-12-22

**Authors:** Joohwan Sung, Sungmin Han, Heesu Park, Hyun-Myung Cho, Soree Hwang, Jong Woong Park, Inchan Youn

**Affiliations:** 1 Center for Bionics, Biomedical Research Division, Korea Institute of Science and Technology, Seoul 02792, Korea; sjh1449@kist.re.kr (J.S.); han0318@kist.re.kr (S.H.); park@kist.re.kr (H.P.); wisjmeng@kist.re.kr (H.-M.C.); srhwang@kist.re.kr (S.H.); 2 Department of Biomedical Science, College of Medicine, Korea University, Seoul 02841, Korea; 3 Department of Artificial Intelligence, Korea University, Seoul 02841, Korea; 4 School of Biomedical Engineering, Korea University, Seoul 02841, Korea

**Keywords:** inertial measurement unit, wearable sensor, gait analysis, deep neural network, long short-term memory

## Abstract

The joint angle during gait is an important indicator, such as injury risk index, rehabilitation status evaluation, etc. To analyze gait, inertial measurement unit (IMU) sensors have been used in studies and continuously developed; however, they are difficult to utilize in daily life because of the inconvenience of having to attach multiple sensors together and the difficulty of long-term use due to the battery consumption required for high data sampling rates. To overcome these problems, this study propose a multi-joint angle estimation method based on a long short-term memory (LSTM) recurrent neural network with a single low-frequency (23 Hz) IMU sensor. IMU sensor data attached to the lateral shank were measured during overground walking at a self-selected speed for 30 healthy young persons. The results show a comparatively good accuracy level, similar to previous studies using high-frequency IMU sensors. Compared to the reference results obtained from the motion capture system, the estimated angle coefficient of determination ($R^{2}$) is greater than 0.74, and the root mean square error and normalized root mean square error (NRMSE) are less than 7° and 9.87%, respectively. The knee joint showed the best estimation performance in terms of the NRMSE and $R^{2}$ among the hip, knee, and ankle joints.

## 1. Introduction

Gait is the basic method of movement, and gait parameters contain much biomechanical information. Among various gait parameters, the lower limb joint (hip, knee, and ankle) angle on the sagittal plane is relevant to the clinical and physical condition of the human. Multi-joint angle information can serve as indicators of an injury risk index in the elderly [1,2,3], the effect of rehabilitation for total knee replacement surgery patients [4,5], and the gait state and running ability of humans [6,7]. Motion capture (MoCap) systems are widely used for accurate gait analysis in the laboratory [8,9]; markers are attached to each subject according to the marker set, and the gait is then analyzed by creating a model based on the marker positions. However, this instrument is difficult to use in a daily life environment because of its high cost, long preparation and setup time, and requirement for a large space [10,11]. To overcome these problems, several gait analysis studies have suggested using inertial measurement unit (IMU) sensors as an alternative method because of their efficacy, detachability, non-contact nature and lower cost [12,13,14,15]. Unfortunately, most studies in this field have still encountered some problems in terms of using them for gait analysis in everyday life. First, two or multiple IMU sensors can be attached to the lower extremities or back and chest to calculate joint angles. As more sensors are attached, we can expect higher accuracy and more degrees of freedom in terms of estimating joint angles [16]. However, it is impractical to measure multiple joint angles during gait using multiple sensors because of the long time required for preparation and the inconvenience to daily life. Second, in several studies, experiments for developing human gait analysis algorithms have been conducted on a treadmill rather than over the ground [17,18]. According to previous studies, walking consistently at the same speed on a treadmill produces a constant walking pattern rather than walking overground [19,20,21]. Since the speed and pattern of walking outside are diverse, the walking analysis experiment should be conducted under the corresponding conditions. The third problem is power consumption, with a high sampling rate resulting in high power consumption [22,23]. With a high sampling rate, more data is obtained, improving the accuracy of the estimated results [24]. However, high sampling rates require more power from the battery. In addition, high-frequency sensors are relatively high-cost pieces of equipment and require large batteries for long-term use, thus difficult to apply in daily life.

To address these considerations, this paper proposes a novel multi-joint angle estimation algorithm using a single low-frequency IMU sensor. We attached a single IMU sensor to the lateral shank of the right lower limb considering the convenience of the user in terms of simply adding functionality to existing exercise tools. Strap bands are used to support the patella or relieve knee pain, the efficacy of which has been proven in many previous studies [25,26]. If the IMU sensor is added to an existing exercise strap band, the user will not experience any discomfort or resistance when using it in daily life. To reproduce the situation of walking outdoors as much as possible in the laboratory, we conducted all walking tests overground instead of on a treadmill. To overcome the limitation of performing gait analysis with a single sensor at a low sampling rate, we applied the long short-term memory (LSTM) algorithm, which is a powerful model for time series data. Furthermore, the feature selection method was used to find the optimized input features based on their contribution to the regression, increasing the accuracy of the model estimation performance. We evaluated the performance of the proposed model by calculating the root mean square error (RMSE), normalized root mean square error (NRMSE), and coefficient of determination ($R^{2}$), which are widely used to assess estimation results. The results of this study have future implications for gait analysis in daily life.

## 2. Materials and Methods

### 2.1. Experimental Equipment and Protocol

In all, 30 healthy male young participants were recruited for the collection of gait data: age, 23.5 ± 2.5 years; height, 173.4 ± 5.8 cm; body mass, 72.4 ± 10 kg; and BMI, 24 ± 2.8 kg/m^2^. Written consent was obtained from each participant prior to the experiment, and all experiments were conducted in strict accordance with the Korea Institute of Science and Technology (KIST) Ethics guidelines (IRB-2019-027).

**Figure 1:**
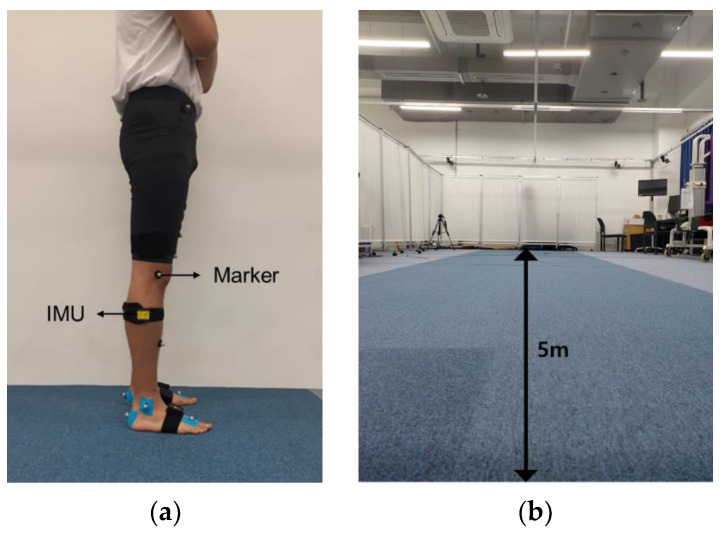
Experimental setup. (**a**) Markers for motion tracking and the inertial measurement unit (IMU) attached on the lateral shank of the right side. (**b**) Straight 5-m-long course with 10 MoCap cameras for overground walking.

While walking, joint angles on the sagittal plane were calculated using a single IMU sensor (eCEN, Eburnean, Seoul, Korea) with an acceleration resolution of 0.01 g and an angular velocity resolution of 0.05°/s. The size of the IMU sensor was 51.3 × 36 × 15 mm^3^, and the mass of the IMU sensor was 10 g. The IMU sensor collected data at a sampling frequency of 23 Hz and used BLE 4.0 for wireless transmission. The IMU sensor output consisted of 3-axis acceleration, angular velocity, and attitude angle data. The measuring range for acceleration was ±16 g; angular velocity, ±2000°/s; and angle, X and Z ± 180° and Y ± 90°. As shown in Figure 1a, the IMU sensor was attached to a lateral shank of the right side parallel to the sagittal plane (the YZ plane in the global frame), which is a flat surface at a distance of approximately 10 cm from the femur lateral epicondyle. As the gold standard for obtaining joint angles, a 10-camera optical MoCap system (Motion Analysis, CA, USA) was used. Nineteen reflective markers were attached to the participant’s sacrum, pelvis, thigh, knee, shank, ankle, toe, and heel according to a Helen Hays marker set [27,28]. The MoCap system samples 60 frames per second. As a result, marker-based calculated gait kinematic data are also 60 Hz.

Thirty participants walked at their preferred walking speed eight times across a 5-m-long walkway, as shown in Figure 1b. The average walking speed of the participants was 1.27±0.13 m/s, and the average cadence and stride length of the participants were 113.87±7.32 steps/min and 1.33±0.10 m, respectively. Since successive measurements result in similar gait speeds and patterns, we provided a one-minute break between trials.

### 2.2. Data Preprocessing

#### 2.2.1. Data Resampling and Filtering

Data analysis was carried out in the Python programming environment. As a result of gait analysis, a total of 483 cycles were obtained from 30 participants. Every marker data point was filtered with a 4th-order Butterworth low-pass filter with a cutoff frequency of 4 Hz. The IMU sensor data consist of nine different values for the 3-axis angle, angular velocity and acceleration data. The 3-axis angle is represented as pitch, roll, and yaw. The IMU data were smoothed by a median filter using a 5-sample window. The IMU signal length of every gait cycle was different among participants and among trials for each participant. To match the size of the kinematic data calculated from MoCap and the IMU samples of each gait cycle, all kinematic samples obtained from MoCap marker data were resampled to the IMU sample size corresponding to each gait cycle.

#### 2.2.2. Feature Extraction

Deep learning-based feature selection techniques have been widely applied in motion analysis to improve estimation accuracy and overcome the limitation of the slow training rate [16,29]. Feature selection is the process of reducing the number of noninformative or redundant predictors to increase predictive model performance. Features that are not relevant to the target variable can act as noise and degrade the performance of a model. Among the many feature selection methods, we selected the filter feature selection method, which uses statistical techniques (Pearson’s correlation coefficient) to evaluate the relationship between each input variable and the target variable [30]. The feature selection process was performed separately for each joint, and the effectiveness of feature selection was proven by comparing the result obtained with selected subset features to the result obtained with all features. For each joint, every feature that showed a meaningful correlation value with the label was selected to obtain a multi-joint estimation angle with just one calculation.

### 2.3. Deep Learning Model

#### 2.3.1. Background

The recurrent neural network (RNN) model processes inputs and outputs as a sequence unit, which is suitable for processing sequential data, such as sound and text [31,32,33,34]. The hidden state that is updated based on previous and current information from sequential data allows RNNs to perform better than other general multilayer perceptron neural network models. The architecture of an RNN consists of the following equations. To obtain the hidden state value at the current time t as $h_{t}$, we need two weights, $W_{hh}$ and $W_{xh},$ and a bias $b_{h}$, shown in Equation (1) below. The weight $W_{xh}$ is for the current input value $x_{t}$, and the weight $W_{hh}$ is for the previous hidden state $h_{t-1}$. Subsequently, the output $y_{t}$ is calculated through the obtained $h_{t}$, $W_{hy}$ and bias $b_{y}$, as shown in Equation (2) below. As a result, previous information in time series data influences the current neural network calculation.

(1) $$h_{t}=tanh\left(W_{hh}h_{t-1}+W_{xh}x_{t}+b_{h}\right)$$

(2) $$y_{t}=W_{hy}h_{t}+b_{y}$$

However, the basic RNN architecture has limitations, called vanishing and exploding gradients, in terms of long-term dependency [35], which means that the gradient becomes too small or too large to train properly during RNN backpropagation. To solve these problems, the LSTM algorithm was developed by Hochreiter & Schmidhuber [36]. By using cell state $C_{t}$, LSTM prevents backpropagated errors from vanishing or exploding [37]. The forget gate value $f_{t}$ determines what information to discard from the cell state. In the next step, the input gate value $i_{t}$ determines which of the incoming new information to store in the cell state. Finally, output gate $o_{t}$ determines which part of the cell state to send. In short, the LSTM algorithm solves the problem of basic RNNs by training the model to store important input and delete less important input in a long-term state. The equations are as follows, where W and b denote weight and bias, respectively.

#### 2.3.2. Model Design

**Figure 2:**
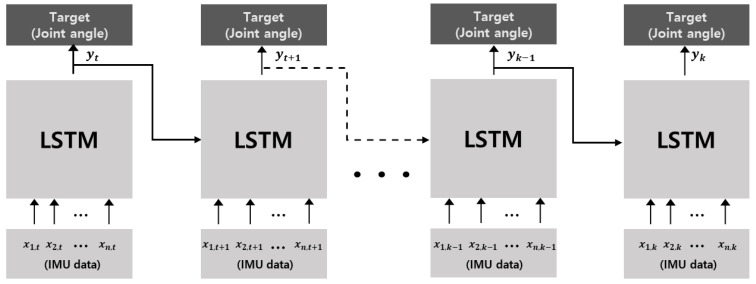
Architecture of the LSTM model, where $x_{t}$ and $y_{t}$ denote the preprocessed IMU input data and multi-joint angles as output from the LSTM deep neural network. In time sequence data, the output at time step t, $y_{t}$ affects the result at time step t+1 as the input. While repeating this procedure as training, the cell state determines which information to remember and which to forget to improve performance.

We propose a deep neural network based on the LSTM model to estimate multi-joint angles. The network structure of the LSTM model has 1 layer with extracted feature inputs. The batch size for the training model was selected to be 256, and the hidden size was fixed to 10. We utilized Adam as an optimizer to train the proposed model [38]. Adam is an algorithm for stochastic gradient descent for training deep neural network models. The length of the input data sequence was set to 5, and the number of epochs was 500 based on loss convergence in the training process. Finally, we used the hyperbolic tangent (Tanh) as an activation function, which is commonly used in RNN or LSTM networks. Table 1 shows the list of hyperparameters used in this model, and Figure 2 shows the structure of the proposed model.

The MSE is one of the most commonly used loss functions for regression. The loss function is the summation of the mean of squared difference data between the true and predicted values for each joint and can be written as follows:

(3) $$\mathrm{MSE}=A\times \frac{1}{N}\overset{N}{\underset{i=1}{\sum }}{\left(y_{i,Hip}-{\hat{y}}_{i,Hip}\right)}^{2}\;+B\times \frac{1}{N}\overset{N}{\underset{i=1}{\sum }}{\left(y_{i,Knee}-{\hat{y}}_{i,Knee}\right)}^{2}+C\times \frac{1}{N}\overset{N}{\underset{i=1}{\sum }}{\left(y_{i,Ankle}-{\hat{y}}_{i,Ankle}\right)}^{2}$$

where *N* is the number of samples tested; y and $\hat{y}$ are the gold standard and predicted values, respectively; and A, B, and C are empirically selected to be 3, 1, and 4, respectively. We performed a normalization process to scale the input features to a range of 0 to 1 as follows:

(4) $$X=\frac{X_{origin}-X_{min}}{X_{max}-X_{min}}$$

where X is the normalized value; $X_{origin}$ is the raw value; and $X_{max}$ and $X_{min}$ denote the maximum and minimum original values, respectively. Normalization reduced the internal covariate shift to improve training and prevent overfitting. After normalization, the IMU data were applied to the LSTM model as input data to estimate the angle of each joint, as shown in Figure 2.

### 2.4. Data Analysis

To quantitatively measure the joint angle prediction performance using the LSTM model, we used the RMSE, $R^{2}$, and the NRMSE. The NRMSE is normalized by dividing the difference between the maximum and minimum values of the prediction result using the LSTM model for each gait cycle. The RMSE and NRMSE values represent the error of a model predicting quantitative data, and the $R^{2}$ value represents the goodness of fit of the prediction result. The equations to calculate the RMSE, NRMSE and $R^{2}$ are as follows.

(5) $$RMSE=\sqrt{\sum_{i=1}^{n}\frac{{\left({\hat{y}}_{i}-y_{i}\right)}^{2}}{n}},\;NRMSE=\frac{RMSE}{y_{max}-y_{min}},\;R^{2}=1-\frac{\sum_{i}{\left(y_{i}-{\hat{y}}_{i}\right)}^{2}}{\sum_{i}{\left(y_{i}-\bar{y}\right)}^{2}}$$

In the equation for the RMSE, *n* denotes the number of samples, and y and $\hat{y}$ are the gold standard and predicted values, respectively. In the equation for $R^{2}$, y denotes the observed response variable, $\bar{y}$ its mean and $\hat{y}$ the corresponding predicted values [39].

**Figure 3:**
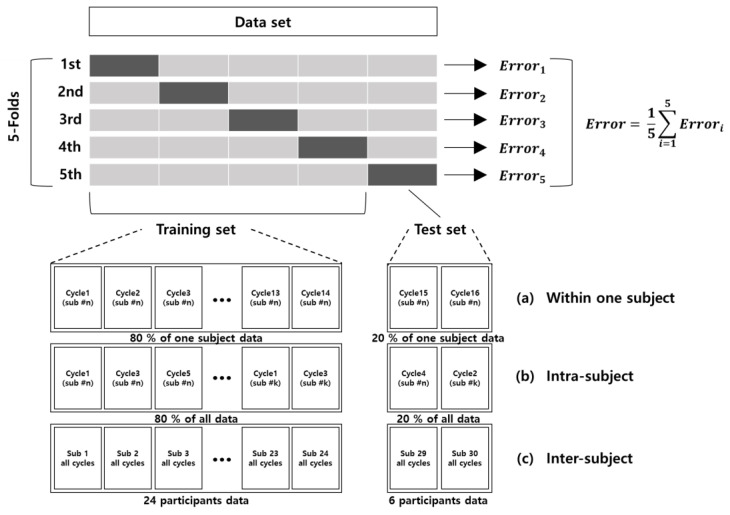
Five-fold cross-validation was applied to evaluate the model. The training data set and test data set were divided in three ways in each fold: (**a**) Within-one-subject; data were split for training and testing within the #n-th subject at a ratio of 8:2. (**b**) Intra-subject; data were split for training and testing from randomly concatenated whole gait cycle data at a ratio of 8:2. (**c**) Inter-subject; all gait cycles from 24 randomly chosen participants were chosen as training data, and all gait cycles from the remaining six participants were assigned as test data.

In this study, we evaluated an LSTM model using all the available features from an IMU sensor. Then, we applied the selected features using the feature extraction method, which removed redundant features. To train and test the LSTM model quantitatively, the training data set and test data set were divided in three different ways: (1) within one subject, (2) intra-subject, and (3) inter-subject. In the within-one-subject method, we divided the training data set and test data set by the gait cycle within one participant. Approximately 20 cycles of overground gait were conducted by each participant; 80% (16 cycles) of the data were divided into a training data set, and 20% (4 cycles) were divided into a test set, as shown in Figure 3a. In the intra-subject method, we combined all 30 participants’ data randomly by gait cycle and then split them at 80% for the training data set and the remaining 20% for the test data set regardless of the consequences of the trials (Figure 3b). In the inter-subject method, the training data set and test data set were divided based on the subject. Twenty-four people, equivalent to 80%, were used for training, and the remaining six people (20%) who had not been involved in the training were used for testing. In each of the three methods, 5-fold cross-validation (CV) was applied to calculate optimal parameters, which performs the fitting procedure a total of five times to avoid biased model performance from the way the training and testing subsets are selected.

## 3. Results

In this section, the results of the feature selection applied to increase the regression accuracy are shown. After that, the results are shown when feature selection is applied by each of the three methods, depending on how the training set and test set are divided.

### 3.1. Feature Extraction

**Figure 4:**
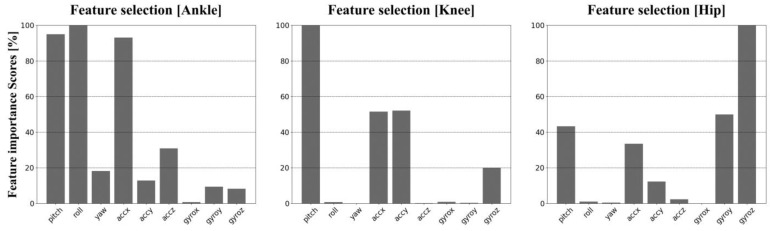
Importance of features in predicting each joint angle, where the *X*-axis denotes features from the IMU data and the *Y*-axis denotes the importance of features divided by the maximum importance value among features.

Figure 4 shows the degree of importance of the features required to predict each joint angle among nine features as a percentage. The selected features are informative for predicting the corresponding joint, and the remaining features are redundant. Through trial and error, features representing more than 30% importance were selected to estimate each joint angle. The selected features for the ankle joint were pitch, roll and *x*-axis acceleration, which are the most importance. The pitch was also shown to be the most important in predicting the knee joint angle, while the *x*-axis and *y*-axis accelerations presented significant importance. Unlike the other two joints, the *z*-axis gyroscope was the most important in estimating the hip joint angle. The pitch, *x*-axis acceleration, and *y*-axis gyroscope also presented a greater than 30% importance.

### 3.2. Estimation of Joint Angles

By using data from the single low-frequency IMU, the joint kinematics during overground gait were predicted based on the LSTM model. As kinematic data, we calculated hip, knee and ankle joint angles in the sagittal plane. The predicted joint kinematic values were compared with the kinematic results from MoCap. We estimated the joint angles with two different subsets as input features. First, data of all nine features obtained from IMU were used as input features. After that, six selected features, consisting of pitch, roll, x- and *y*-axis acceleration, and y- and *z*-axis gyroscope obtained from the feature extraction process at each joint, were used as input features. To evaluate model performance, the training data set and test data set were divided in three different ways: (1) within one subject, (2) intra-subject and (3) inter-subject. Overall, the angles estimated using the selected features as input were better than those estimated using all features when compared to the MoCap results. In all three data division methods, the knee joint angle was estimated more accurately than the hip and ankle joint angles.

**Figure 5:**
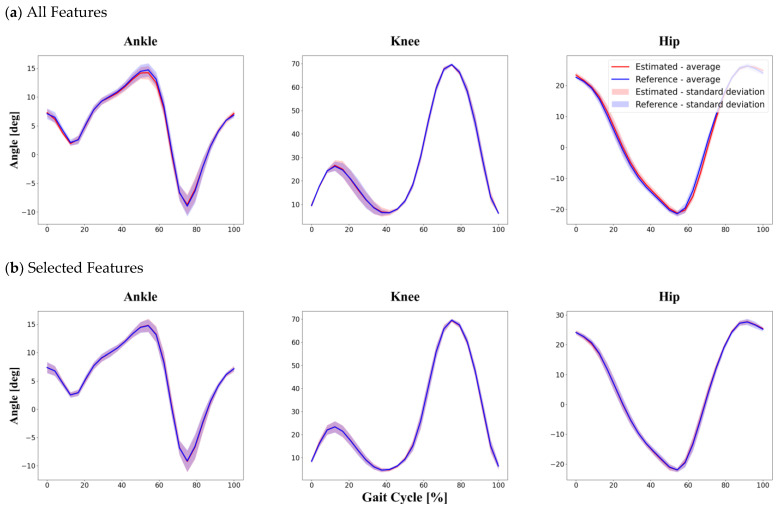
Within-one-subject division method. The graphs show the results of the test set randomly divided into training and test set data at 8:2 from among the total gait cycle data from one participant. Comparison between reference data obtained by a conventional MoCap system and joint angles estimated by the proposed LSTM model using two different feature sets: (**a**) all features as input in the within-one-subject method; (**b**) selected features as input in the within-one-subject method.

The average $R^{2}$ value calculated individually from 30 participants was greater than 0.96 when using all features and 0.98 when using selected features. Moreover, the RMSE and the NRMSE were less than 0.83° and 1.59% when using all features as input and less than 0.47° and 0.91% when using selected features as input. The results show that the method of dividing data within one subject produces the best performance in estimating joint angles compared to the other division methods. Even though the difference was small, we found that the estimation performance improved when using selected features as input. More detailed information is provided in Table 2 and Figure 5 below.

**Figure 6:**
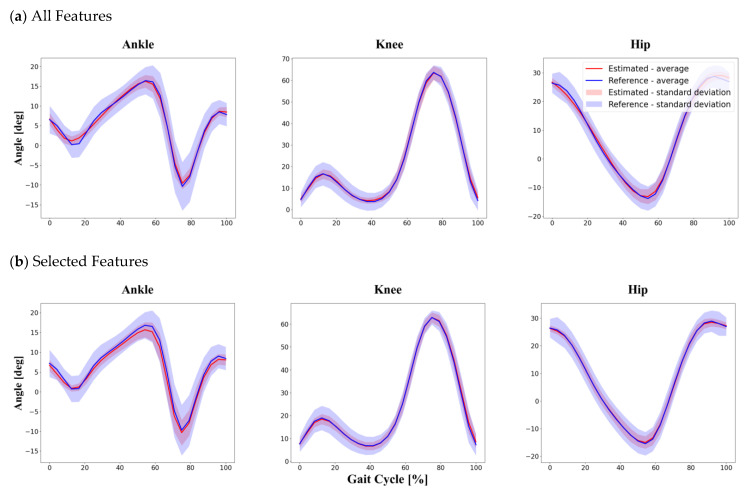
Intra-subject division method. The graphs show the results of the test set randomly divided into training and test set data at 8:2 from among the total gait cycle data from all 30 participants. Comparison between reference data obtained by a conventional Mocap system and joint angles estimated by the proposed LSTM model using two different feature sets: (**a**) all features as input in the intra subject method; (**b**) selected features as input in the intra subject method.

Table 3 and Figure 6 show the comparison between estimated joint angles and corresponding reference joint angles obtained from the MoCap system in the intra-subject section. Among the ankle, knee and hip joints, the knee joint angle obtained using the selected features showed the best accuracy in terms of the $R^{2}$ and NRMSE. When using all features as input to calculate joint angles, the RMSE ranged from 3.96° to 6.34°, and the NRMSE ranged from 8.52% to 9.30%. The RMSE ranged from 3.06° to 5.76°, and the NRMSE ranged from 6.70% to 8.66%, when using selected features as input data.

**Figure 7:**
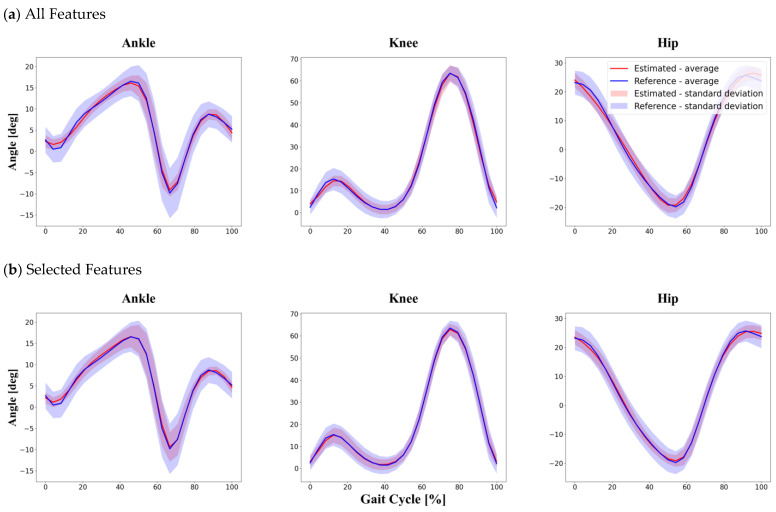
Inter-subject division method. The graphs show the results the entire gait cycle of the six participants corresponding to the test set defined by randomly dividing the 30 participants at a ratio of 8:2. Comparison between reference data obtained by a conventional Mocap system and joint angles estimated by the proposed LSTM model using two different feature sets: (**a**) all features as input in the inter subject method; (**b**) selected features as input in the inter subject method.

Following our hypothesis, the inter-subject method showed the lowest accuracy in predicting joint angles because of testing unseen data sets during the training process. As with other methods of dividing data into training and test sets, knee joint estimation using the selected features as input showed the lowest NRMSE and highest $R^{2}$. With the inter-subject method, the $R^{2}$ was greater than 0.74, and the RMSE and NRMSE were less than 7.00° and 9.87%, respectively. More detailed information is provided in Table 4 and Figure 7 below.

## 4. Discussion

In this work, we proposed an LSTM model for joint angle estimation using a single low-frequency IMU sensor. We conducted the study with one IMU sensor and focused on long-term use to apply the system in our daily lives. The joint angles predicted utilizing the LSTM model showed a reasonably good match with the results obtained using MoCap despite the low number of samples per cycle from the use of a single 23 Hz IMU sensor and a relatively small amount of training data. These results imply that the motion of the shank during walking is an important determinant in calculating multi-joint angles and that the tradeoff between the number of data samples per gait cycle and long-term usage in daily life can be solved by utilizing an LSTM model, which has a powerful deep learning architecture for time series input data. Among the three different training and test data division methods, the within-one-subject method showed the highest accuracy. This is interpreted as the reason for data in the testing set being more similar to data in the trained set within one participant than between subjects. The reason why the inter-subject method showed relatively low performance could be that the LSTM model tested data it had not encountered previously during training. This result enables us to infer that the more we train a model with various gait speeds and patterns, the more accurate the model will become and the wider it can be applied to participants with different gait patterns. Excluding the within-one-subject method, which already showed high accuracy, for the intra- and inter-subject methods, using the selected features as input showed significantly better accuracy than using all features from the IMU sensor as input. This is thought to be because irrelevant input features are removed through feature selection processing, thus finding the optimal subsets to increase model performance using the correlation with the target variable, and only selected features relevant to the target input are used to estimate joint angles. Regarding other previous studies, Moshen et al. [18] proposed a convolutional neural network (CNN) model-based joint angle estimation method using a shoe-mounted accelerometer with a sampling rate of 100 Hz during treadmill walking. Using the inter-subject division method, the hip and ankle joint NRMSEs were found to be 9.9% and 11.1%, respectively. Dorschky et al. [40] proposed kinematic and kinetic estimation methods using dynamic optimization, which yielded a dynamically consistent simulation based on data from accelerometers and gyroscopes in seven IMUs. According to these research results, the hip, knee, and ankle joint relative RMSEs (rRMSEs) were found to be 21.9%, 8.8%, and 10.9%, respectively. Moshen et al. [41] also presented a four-layer CNN model-based joint angle calculation method utilizing strain sensors with a sampling rate of 100 Hz during treadmill walking. The hip and ankle joint NRMSEs were 9.34% and 9.99%, respectively, when the training and test data were divided using the inter-subject method. Among the three joints, the estimated hip and ankle joint angles showed larger errors compared with the MoCap results than did the estimated knee joint angle. We reasoned that because the IMU is attached to the shank, which is closest to the knee joint, it enables the model to predict movement of the knee joint better than that of other joints. Furthermore, knee movement in the sagittal plane is relatively constant between subjects and has a lower degree of freedom than other joints [18].

The limitations of our study and the technical problems to be solved include the calculation of additional gait parameters and the accuracy of estimation angles, the applicability in various gait environments, and the user-customized algorithm. First, when the proposed joint angle prediction method is utilized, the estimated angular error is within the maximum RMSE of $7^{{}^{\circ}}$ and the maximum NRMSE of 9.87%. Although the low accuracy limitation was overcome somewhat by using the LSTM model, the accuracy could not be further improved because of the use of a single IMU with a small sample size due to a low sampling rate to obtain data from overground trials, which allows various gait patterns, simulating walking in daily life. It is expected to have higher accuracy in terms of RMSE and $R^{2}$ if the same model is used with IMU with a higher sampling rate. For example, even if an experiment is conducted at the same self-selected speed, in a slow speed group, more samples may be obtained per gate cycle, so the accuracy may be expected to increase, whereas in a fast speed group, fewer samples may be obtained and thus the accuracy may be decreased. As seen with the intra-subject and within-one-subject methods, improved accuracy can be expected by training big data obtained through more gait experiments with many subjects in the future. In case of high speed such as running, IMU with higher sampling rates needs to be used to calculate joint angles. Second, all experiments were conducted indoors because the MoCap results were used as the gold standard. However, according to previous studies, different gait kinematic results are obtained depending on the test environment, including whether the subjects are barefoot or in shoes [42], indoors or outdoors [43], male or female [44], and young or old [45]. Thus, in future studies, a larger group of participants with differences in demographic features, such as sex, age, height, and weight, needs to be recruited, and experiments should be conducted in various environments. In particular, patients who have undergone knee surgery or have a brain disease show differences in gait characteristics from subjects in the control group [46,47]; by using this fact, it is possible to quantitatively determine the rehabilitation status of patients or the presence of disease [8]. Since the proposed algorithm is the result of trials in 30 healthy young males, if this model is applied directly to the patient, it would not show as good performance as healthy subjects. To overcome this problem, additional experiments in patients are needed in the future study, and it is necessary to confirm the possibility of applying the developed model in this case by utilizing another verified system instead of MoCap. Third, additional gait parameter results, such as cadence, speed, stride length, and step width, were not calculated in this paper. However, this information is needed for extensive gait analysis in terms of assessment or diagnosis. To this end, using participants’ body dimensions, such as the length of the leg and foot, to examine biomechanics based on estimated joint angles it is expected to be useful for obtaining additional gait parameters in further studies. Last, sensitivity of IMU related to attachment location. If the IMU is attached to a different side or other position of the lower limb, it will have completely different accelerometers and gyroscopes for the same motion, resulting in a different performance. To overcome this, in the future study, it is needed to propose algorithms that are not affected by the attachment position of the sensor and can be applied to various attachment positions.

## 5. Conclusions

This paper presented a method that for estimating the hip, knee, and ankle joint angles in the sagittal plane during walking using only a single IMU with a low sampling rate for application outdoors in daily life. Using a 23 Hz sensor could reduce the time taken to collect data and reduce the memory space; however, the estimation accuracy value is limited. Despite the use of data from a shank-mounted IMU with a low sampling rate and the overground test environment resulting in inconsistent gait patterns, the multi-joint angles were well predicted by using the feature selection method and the LSTM model with a relatively small amount of training data. The results indicate that the LSTM algorithm, which is widely used for time series data, is a good deep learning algorithm for estimating human walking behavior. Furthermore, it is especially notable that movement of the shank is a determinant factor in estimating multi-joint angles during overground walking. This reliable predictive performance is expected to play a complementary role in evaluating users’ exercise ability or patients’ rehabilitation status through gait analysis in the future.

## Figures and Tables

**Table 1:** Hyperparameters used in the LSTM model.

Hyperparameters	Values
Number of layers	1
Batch size	256
Hidden size	50
Optimizer	Adam
Learning rate	0.001
Sequence length	5
Number of epochs	500
Activation function	Tanh

**Table 2:** Within-one-subject division method, 5-fold cross-validation comparison between joint angles estimated by the proposed LSTM model using two different input feature sets and a conventional MoCap system.

		Ankle Joint	Knee Joint	Hip Joint
All Features	$R^{2}$	0.96	0.99	0.97
	RMSE (°)	0.42	0.36	0.83
	NRMSE (%)	1.43	0.57	1.59
Selected Features	$R^{2}$	0.98	0.99	0.98
	RMSE (°)	0.14	0.15	0.47
	NRMSE (%)	0.54	0.24	0.91

**Table 3:** Intra-subject division method, 5-fold cross-validation comparison between joint angles estimated by the proposed LSTM model using two different input feature sets and a conventional MoCap system.

		Ankle Joint	Knee Joint	Hip Joint
All Features	$R^{2}$	0.73	0.89	0.90
	RMSE (°)	3.96	6.34	5.47
	NRMSE (%)	8.52	9.30	9.01
Selected Features	$R^{2}$	0.83	0.92	0.90
	RMSE (°)	3.06	5.76	4.80
	NRMSE (%)	7.21	6.70	8.66

**Table 4:** Inter-subject division method, 5-fold cross-validation comparison between joint angles estimated by the proposed LSTM model using two different input feature sets and a conventional MoCap system.

		Ankle Joint	Knee Joint	Hip Joint
All Features	$R^{2}$	0.62	0.87	0.84
	RMSE (°)	5.15	8.14	7.49
	NRMSE (%)	12.2	11.01	10.97
Selected Features	$R^{2}$	0.74	0.89	0.86
	RMSE (°)	4.35	7.00	6.19
	NRMSE (%)	9.87	9.10	9.74

## Data Availability

The data used in this study are available on request from the corresponding author. The data are not publicly available because of participant confidentiality.
